# Supplementary figures and images for: Loss of Katnal2 leads to ependymal ciliary hyperfunction and autism-related phenotypes in mice
Source: PLoS Biol. 2024 May 8;22(5):e3002596. doi: 10.1371/journal.pbio.3002596 (PMC11104772; doi:10.1371/journal.pbio.3002596)

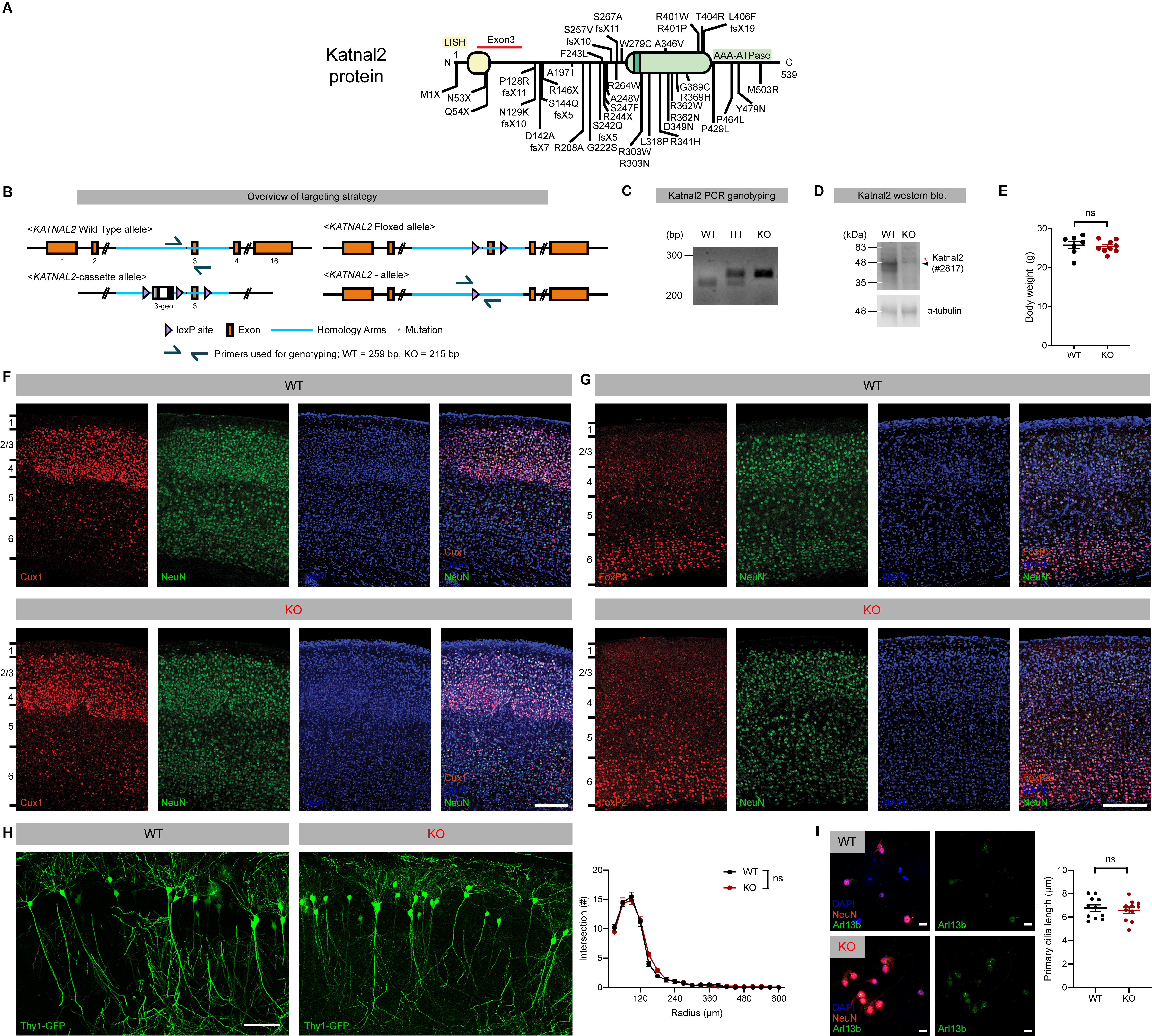

Supplement: S1 Fig — (A) Domain structure of the Katnal2 protein (539 aa-long), known sites of ASD patient-derived point mutations (49 variants from 14 reports), and the protein regions corresponding to exon 3, which was deleted in Katnal2-KO mice (55 aa in the LisH domain). LisH domain, lissencephaly-1 homology domain; AAA-ATPase domain, AAA family ATPase domain. (B) Katnal2 knockout (KO) strategy in mice. The same primer sets are used to detect WT and mutant PCR products (259 and 215 base pairs); they differ by the presence or absence of exon 3, deletion of which leads to a shift in the open-reading frame. (C) PCR genotyping for WT, heterozygous Katnal2-KO (HT), and homozygous Katnal2-KO (KO) mice (postnatal day [P56]). (D) Validation of Katnal2 KO by immunoblot analysis; we used mouse testis samples (P56) instead of brain samples because the expression levels of Katnal2 protein are much greater in the testis relative to the brain. In the brain, Katnal2 expression is confined to select brain regions and cell types, such as ependymal cells lining ventricular walls, as shown by X-gal staining (see Figs 2A and S5). Note also that there is a major Katnal2 protein band (approximately 45 KDa; arrowhead) in WT but not KO testis samples, as revealed by immunostaining with Katnal2 polyclonal antibodies (#2817) raised against aa 517–539 of the Katnal2 protein. Asterisk indicates a nonspecific band recognized by Katnal2 antibodies. (E) Normal body weights in Katnal2-KO mice (2 months). (n = 7 mice [WT], 9 mice [KO], Student’s t test). (F and G) Normal superficial (layer 2/3) and deep (layer 6) cortical layer structures in the Katnal2-KO brain (P56), as revealed by double immunofluorescence staining for NeuN (a neuronal marker) and Cux1 (layer 2/3 marker) or for NeuN and FoxP2 (layer 6). DAPI staining was performed for nuclear staining. The examples shown here are from the somatosensory cortex (layers 1–6). (H) Sholl analysis of CA1 hippocampal neurons from WT and Katnal2-KO mice, visualized by c [file pbio.3002596.s001.tif]

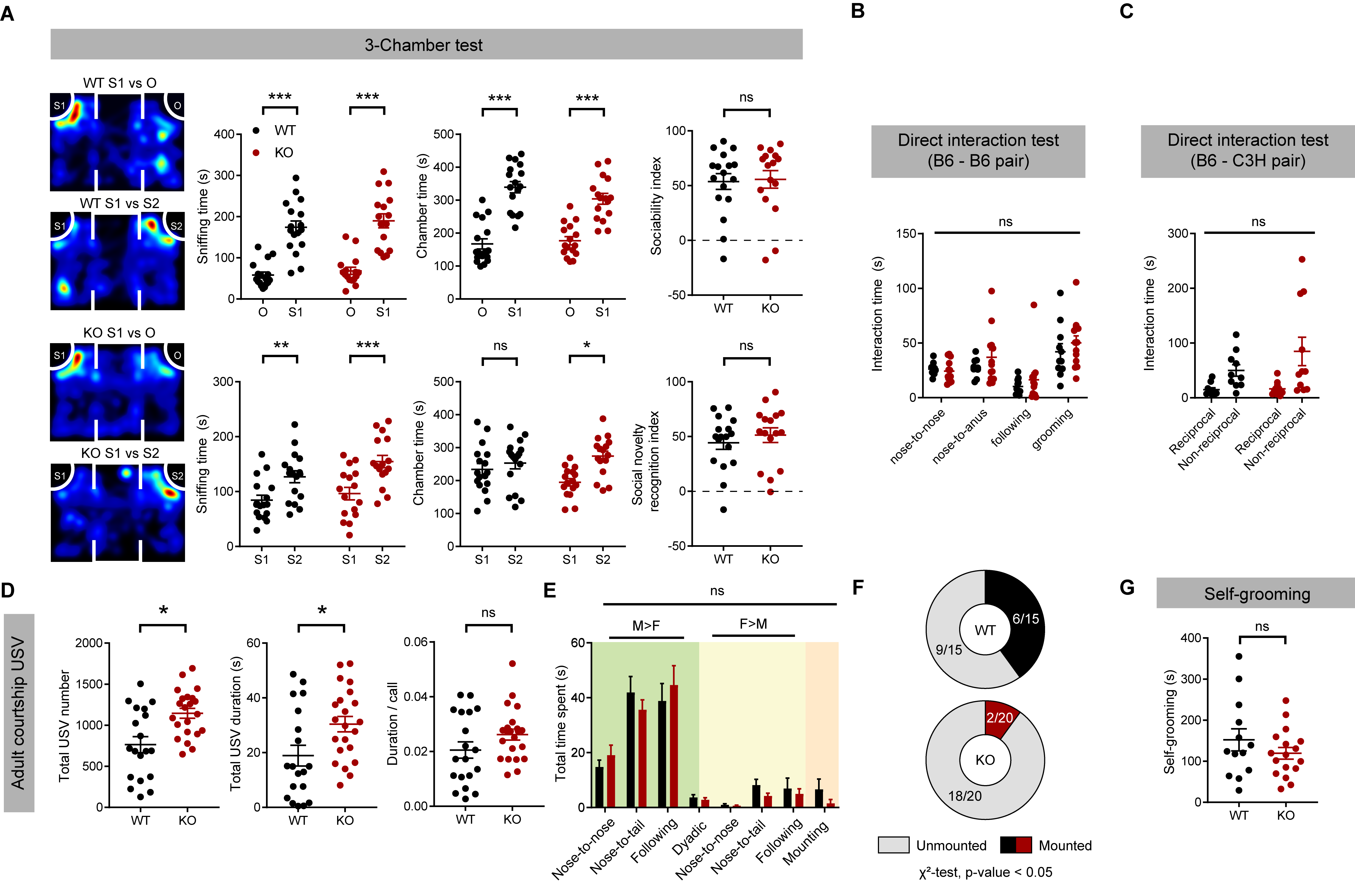

Supplement: S2 Fig — (A) Normal levels of social approach and social novelty recognition in Katnal2-KO mice (2–3 months; male) in the three-chamber test, as shown by time spent sniffing social and object targets (S1/S2, old/new social target; O, object), time spent in the chamber, and the preference index (time difference for S1–O [S2 –S1] / total time × 100). (n = 17 mice [WT], 16 [KO], two-way RM-AVOVA [sniffing time, chamber time], Student’s t-test [sociability index, social novelty recognition index]). (B) Normal levels of direct social interaction in Katnal2-KO mice (2–3 months) in the direct social interaction test, wherein freely moving WT/mutant mouse pairs were used to measure nose-to-nose, nose-to-tail, and following. (n = 11 pairs [WT], 13 [KO], two-way RM-ANOVA). (C) Normal levels of social interaction in Katnal2-KO mice (2–3 months) in a modified version of the direct social interaction test, where a subject mouse interacted with a stranger mouse of C3H background (i.e., with a different coat color) and unidirectional and bidirectional/reciprocal social interactions were measured. (n = 10 mice [WT], 11 [KO], two-way RM-ANOVA). (D) Increased courtship USVs upon encountering a novel female stranger mouse is seen for Katnal2-KO male mice (2–3 months), as indicated by USV call frequency and duration (total and each call). (n = 19 [WT], 22 [KO], Student’s t test [frequency, each-call duration], Mann–Whitney test [total duration]). (E) Largely normal social interactions during the courtship tests, except for mounting, as shown by analysis of male-to-female social interactions (nose-to-nose, nose-to-tail, following), female-to-male interactions (nose-to-nose, nose-to-tail, following), bidirectional interactions (dyadic), and mounting. Mounting behaviors alone show a genotype-related difference, as determined by Student’s t test. Two-way ANOVA encompassing all behaviors did not reveal a genotype-related difference. (n = 12 [WT], 14 [KO], two-way RM-ANOVA). (F) Decreased mounting su [file pbio.3002596.s002.tif]

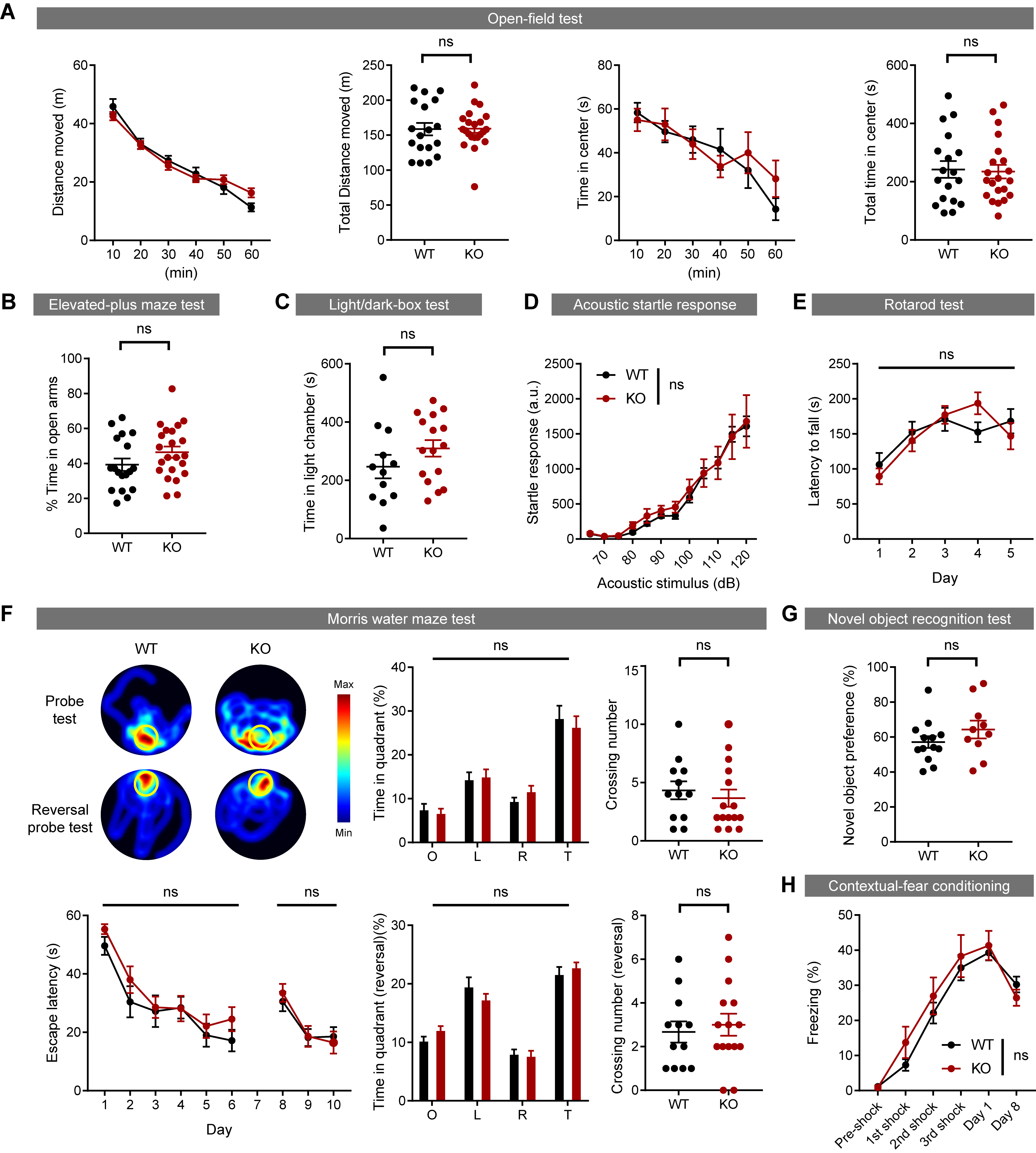

Supplement: S3 Fig — (A) Normal levels of locomotor activity in the open-field test for Katnal2-KO mice (2–3 months), as shown by distance moved. Note that there is no genotype-related difference in the time spent in the center region of the open-field area, suggestive of normal anxiety-like behavior in the mutant mice. (n = 18 mice [WT], 21 [KO], two-way RM-ANOVA [distance moved and time in center], Mann–Whitney test [total distance moved], Student’s t test [total time in center]). (B) Normal levels of anxiety-like behavior in the elevated plus-maze for Katnal2-KO mice (2–3 months), as shown by % time spent in open arms. (n = 18 [WT], 22 [KO], Student’s t test). (C) Normal levels of anxiety-like behavior in the light-dark test for Katnal2-KO mice (2–3 months), as shown by time in the light box. (n = 12 [WT], 16 [KO], Student’s t test). (D) Normal levels of acoustic startle for Katnal2-KO mice (2–3 months). (n = 9 [WT], 7 [KO], two-way RM-ANOVA). (E) Normal levels of motor coordination and learning in the rotarod test in Katnal2-KO mice (2–3 months), as shown by the latency to fall. (n = 17 [WT], 15 [KO], two-way RM-ANOVA). (F) Normal levels of learning and memory in the forward and reversal phases of the Morris water maze test in Katnal2-KO mice (2–3 months). (n = 12 [WT], 15 [KO], two-way RM-ANOVA [escape latency, time in quadrant in probe test and reverse probe test], Mann–Whitney test [crossing number], Student’s t test [crossing number (reversal)]). (G) Normal levels of object recognition memory in the novel object recognition test in Katnal2-KO mice (2–3 months), as shown by the novel-object preference (% time spent exploring the novel object). (n = 13 [WT], 10 [KO], Student’s t test). (H) Normal levels of learning and memory in the contextual fear memory test in Katnal2-KO mice (2–3 months), as shown by freezing levels during fear acquisition, at 24-h retrieval, and at subsequent 8-day retrieval. (n = 18 [WT], 15 [KO], two-way RM-ANOVA). Data values represent means ± SEM. Signifi [file pbio.3002596.s003.tif]

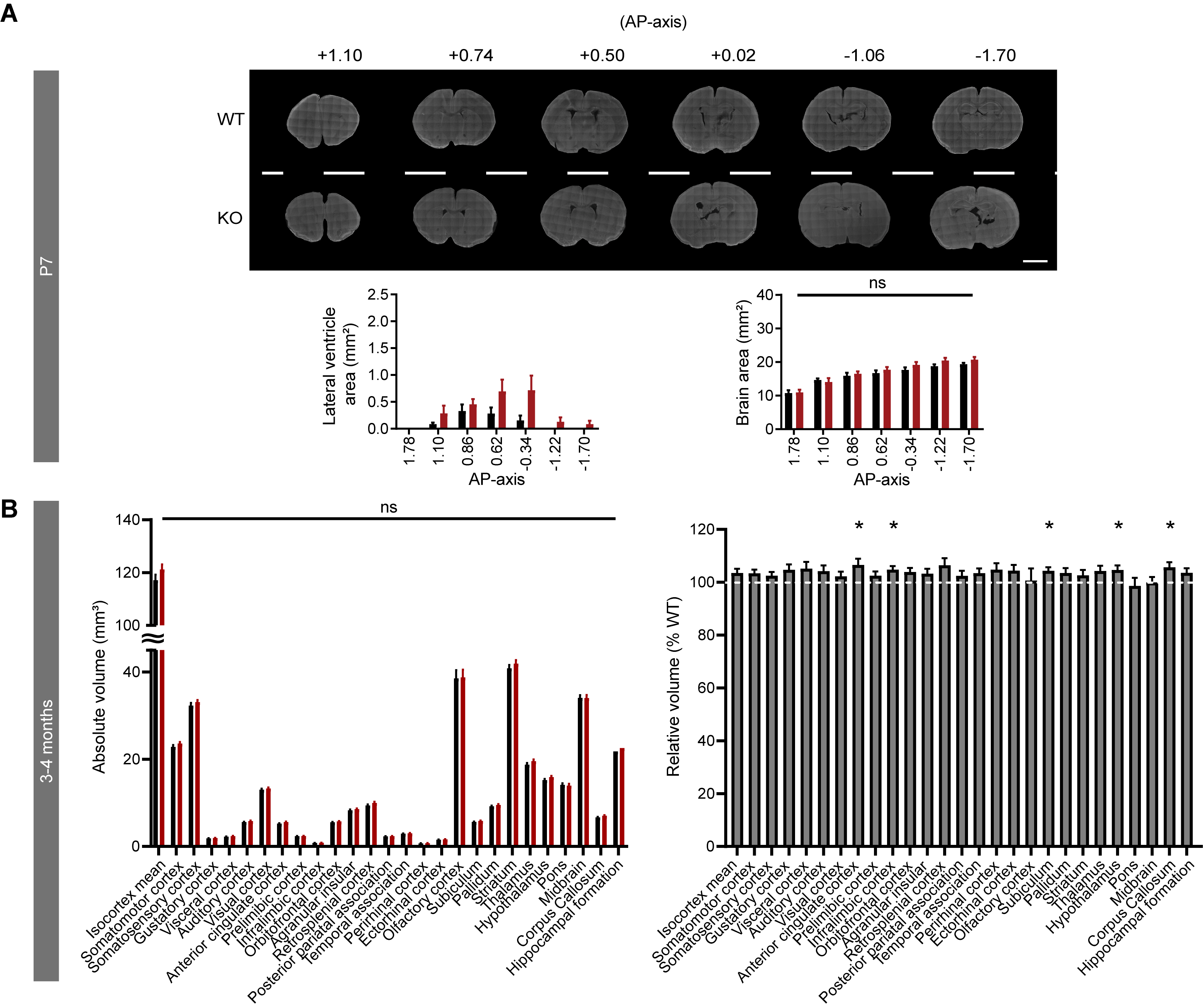

Supplement: S4 Fig — (A) Moderately increased areas of lateral ventricles in Katnal2-KO mice at P7, as shown by measurements derived from coronal brain slices. Note that brain areas are not increased at P7. AP axis, anterior-posterior axis. Scale bar, 2 mm. (n = 5 mice [WT], 6 [KO], two-way RM-ANOVA). (B) Largely normal intracranial brain volumes, with very moderate increases in select brain regions, among Katnal2-KO mice (3 months), as shown by MRI analyses of absolute and relative brain volumes (two-way ANOVA). Note, however, that there are moderate Katnal2-KO-dependent increases some brain regions (one-sample t test). (n = 8 mice [WT], 6 [KO], two-way RM-ANOVA and one-sample t test). Significance is indicated as * (<0.05) or ns (not significant). Statistical results and numerical data values can be found in S1 Data. (TIF) [file pbio.3002596.s004.tif]

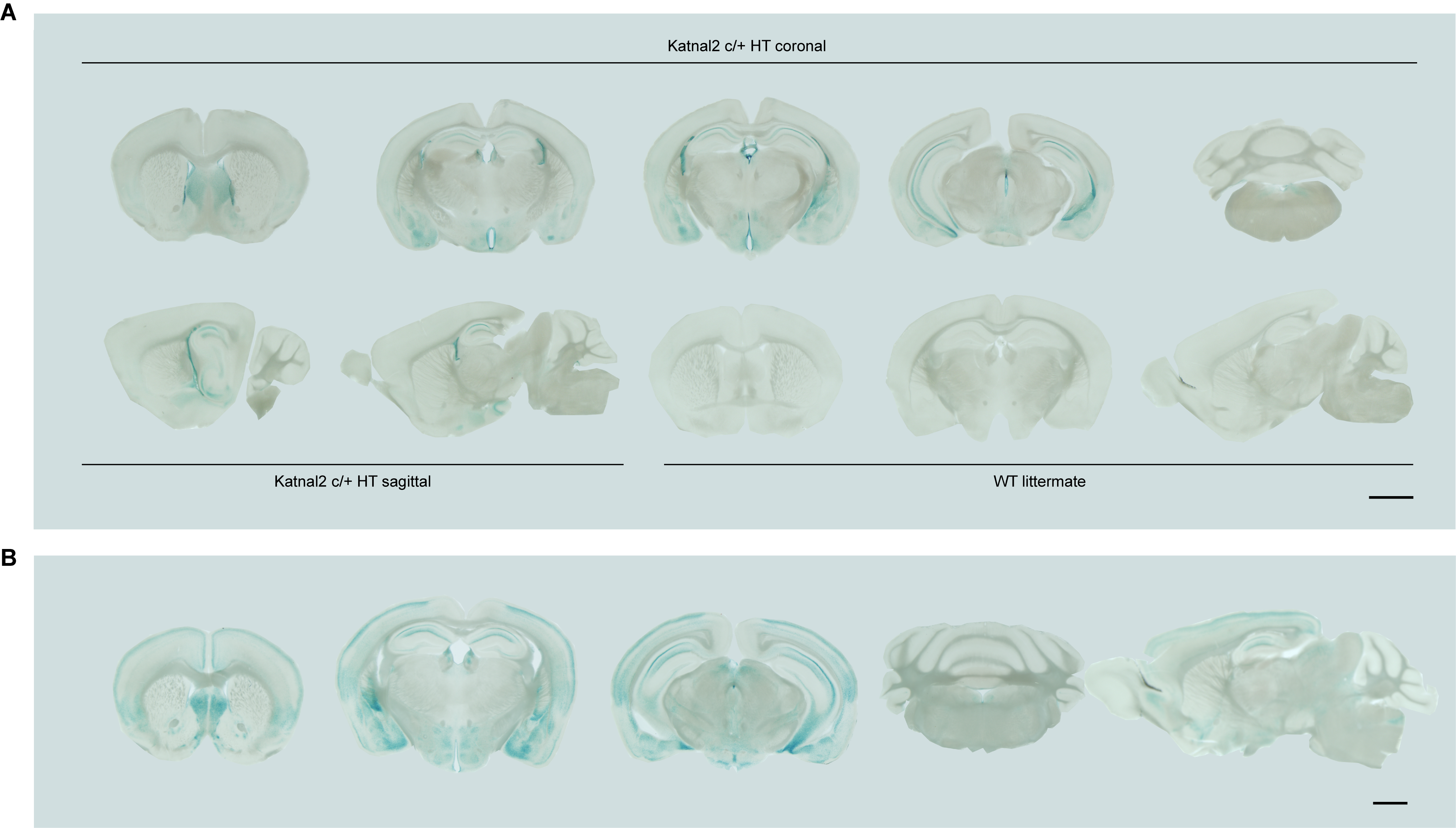

Supplement: S5 Fig — (A and B) Distribution patterns of Katnal2 proteins in the mouse brain, as revealed by X-gal staining of Katnal2-β-galactosidase fusion proteins expressed in Katnal2-KO mice (P21 and P56) with the β-geo cassette unremoved. Scale bar, 1 mm. (TIF) [file pbio.3002596.s005.TIF]

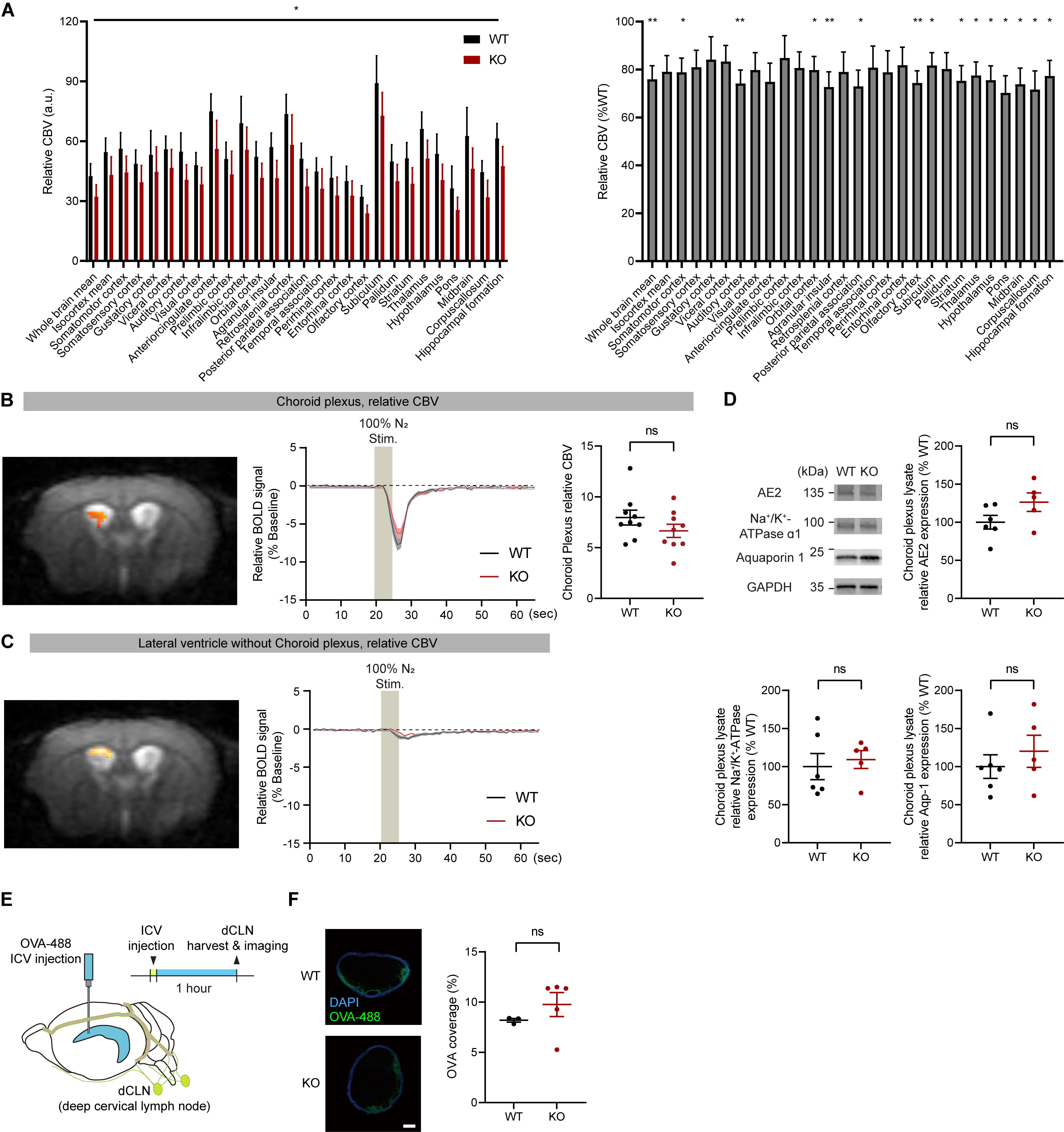

Supplement: S7 Fig — (A) Comparisons of cerebral blood volumes (CBVs) in WT and Katnal2-KO brains, as shown by the extents of decreases in fMRI signals induced by hypoxic nitrogen stimulus in different brain regions (left, relative CBVs) and the KO values normalized to WT values (right, %WT). Iso-cortex includes somatomotor area, somatosensory area, gustatory area, visceral area, auditory area, visual area, anterior cingulate area, prelimbic area, infralimbic area, orbital area, agranular insular area, retrosplenial area, posterior parietal association, temporal association area, perirhinal area, and ectorhinal area. (n = 8 mice [WT], 6 [KO], two-way ANOVA and one-sample t test). (B) Comparable levels of relative CBVs (BOLD signals) in lateral ventricles containing the choroid plexus region in WT and Katnal2-KO mice, as assessed by hypoxic nitrogen stimulus. The red-colored areas indicate representative choroid plexus-containing voxels that we used for the signal tracing. (n = 9 mice [WT], 9 [KO], Mann–Whitney test). (C) Minimal levels of relative CBVs (BOLD signals) in lateral ventricles without the choroid plexus region in WT and Katnal2-KO mice, indicating that the majority of BOLD signals in lateral ventricles are from the choroid plexus and that the effect of the CSF-containing region on BOLD signals is minimal. The yellow-colored areas indicate representative choroid plexus-non-containing lateral ventricle voxels that we used for the signal tracing. (D) Lack of genotype differences in the levels of ion co-transporters (Anion Exchanger 2/AE2 and Na+/K+-ATPase subunit α1) and water channels (aquaporin-1/AQP-1 Student’s t-test), as shown by immunoblot analysis of choroid plexus lysates from WT and Katnal2-KO mice (n = 6 samples [WT], 5 [KO]; 4 choroid plexus samples from 2 mice were pooled to make n of 1). (E) Experimental scheme for the measurement of CSF drainage. Fluorescent ovalbumin proteins (OVA-488) were introduced by intracerebroventricular (icv) injection into the lateral ve [file pbio.3002596.s007.tif]
