# Supplementary material for: Loss of Katnal2 leads to ependymal ciliary hyperfunction and autism-related phenotypes in mice
Source: PLoS Biol. 2024 May 8;22(5):e3002596. doi: 10.1371/journal.pbio.3002596 (PMC11104772; doi:10.1371/journal.pbio.3002596)
Supplement: S1 Raw Images — (PPTX) [file pbio.3002596.s014.pptx]

## Slide 1
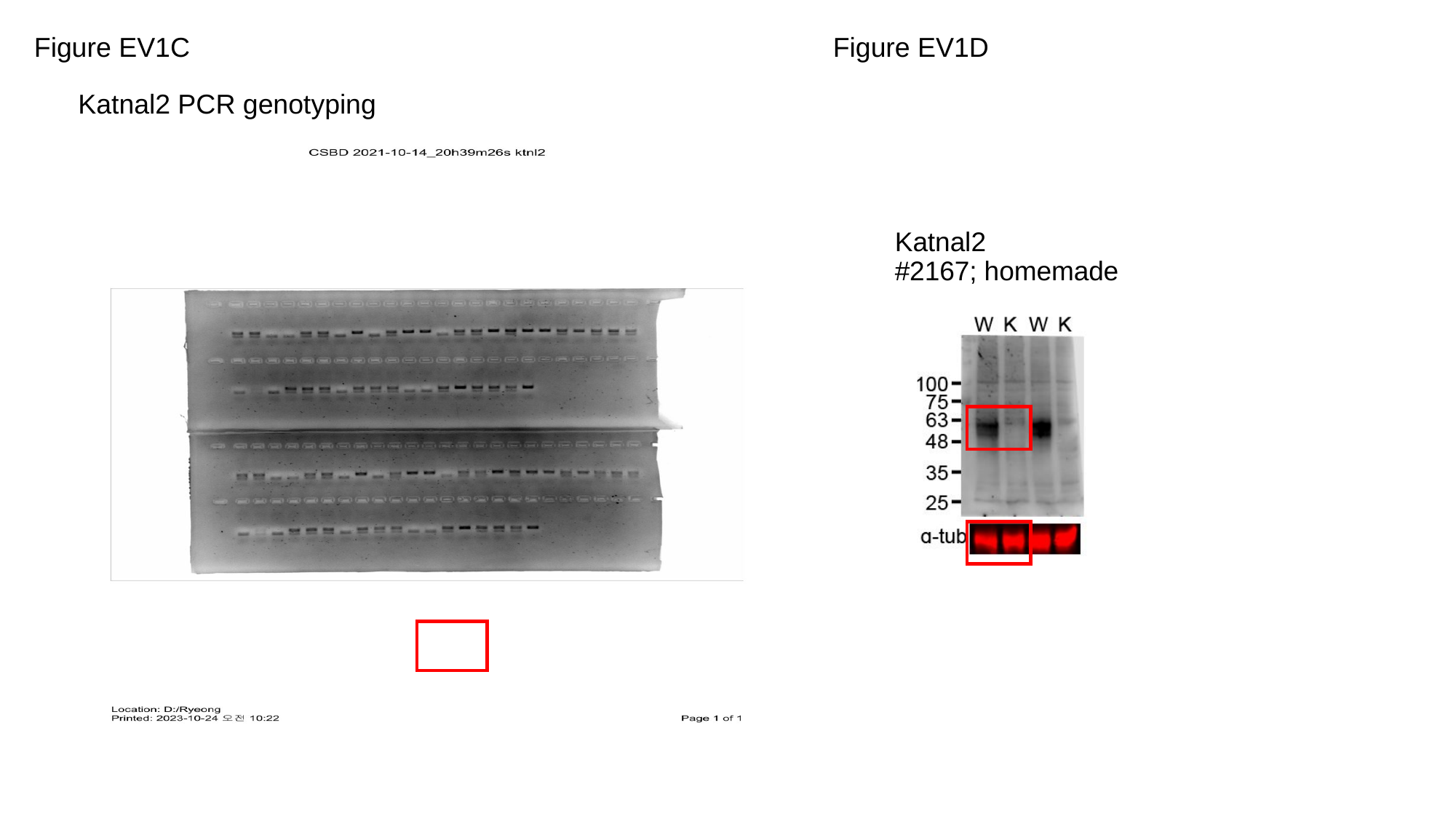

Figure EV1C
Figure EV1D
Katnal2 PCR genotyping
Katnal2
#2167; homemade

## Slide 2
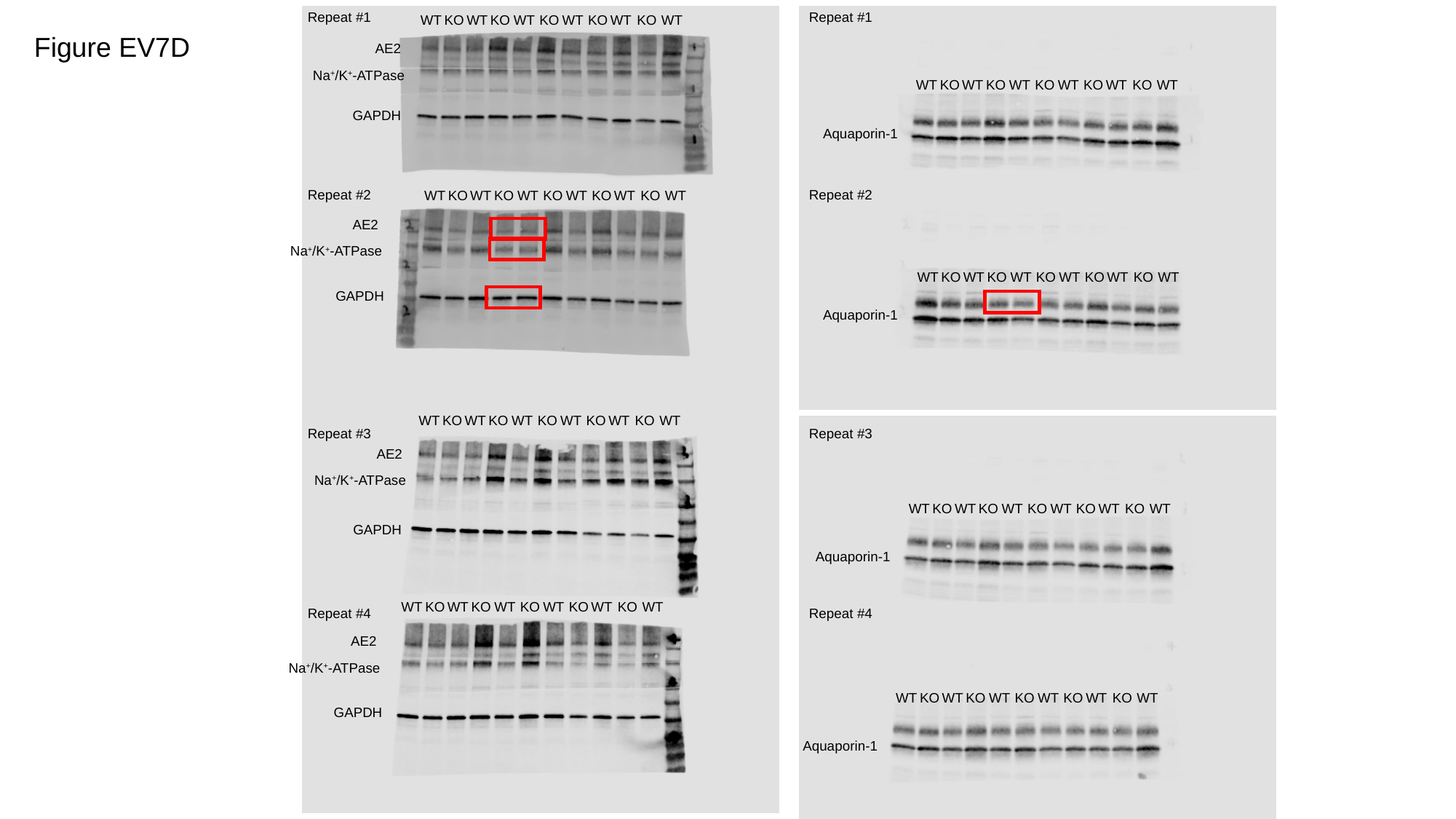

Repeat #1
Repeat #1
WT
KO
WT
KO
WT
KO
WT
KO
WT
KO
WT
Figure EV7D
AE2
Na+/K+-ATPase
WT
KO
WT
KO
WT
KO
WT
KO
WT
KO
WT
GAPDH
Aquaporin-1
Repeat #2
Repeat #2
WT
KO
WT
KO
WT
KO
WT
KO
WT
KO
WT
AE2
Na+/K+-ATPase
WT
KO
WT
KO
WT
KO
WT
KO
WT
KO
WT
GAPDH
Aquaporin-1
WT
KO
WT
KO
WT
KO
WT
KO
WT
KO
WT
Repeat #3
Repeat #3
AE2
Na+/K+-ATPase
WT
KO
WT
KO
WT
KO
WT
KO
WT
KO
WT
GAPDH
Aquaporin-1
WT
KO
WT
KO
WT
KO
WT
KO
WT
KO
WT
Repeat #4
Repeat #4
AE2
Na+/K+-ATPase
WT
KO
WT
KO
WT
KO
WT
KO
WT
KO
WT
GAPDH
Aquaporin-1
