# Supplementary material for: Loss of Katnal2 leads to ependymal ciliary hyperfunction and autism-related phenotypes in mice
Source: PLoS Biol. 2024 May 8;22(5):e3002596. doi: 10.1371/journal.pbio.3002596 (PMC11104772; doi:10.1371/journal.pbio.3002596)
Supplement: Attachment — Submitted filename: Point by point response Kang et al.docx [file pbio.3002596.s016.docx]

**Point-by-point responses to referee’s comments**

**Re: PBIOLOGY-D-23-00657R1**

**Reviewer #1:**

*Katanin, a microtubule-severing ATPase, is a protein complex composed of the regulatory subunit Katanin P80 (KATNB1), the catalytic subunit Katanin P60 (KATNA1), and two additional Katanin P60-like proteins (KATNAL1 and KATNAL2). A recent study of Katnal1 found that homozygous missense mutations in Kantal1 caused ventriculomegaly due to abnormally long ependymal cilia with impaired motility (Banks et al., 2018). In this study, the authors claim that Katnal2 KO also results in increased length of ependymal cilia; however, the motility of ependymal cilia is enhanced, raising hydraulic pressure within the ventricular system to cause ventriculomegaly. Although ventriculomegaly and hydrocephalus have been previously described in mutation of other Katanin family members, such as Katnal1 and Katnb1, a strength of this manuscript is that Katnal2 mutation has not yet been linked with ventriculomegaly. However, major weaknesses of this manuscript are that the data and
analyses do not support their hypothesis and claims on the mechanism of ventriculomegaly. Due to this, my recommended course of action is rejection of this manuscript.*

🡪 We appreciate the helpful comments of the reviewer and admit some weak aspects of this manuscript. However, we tried our best to address the review comments by performing additional experiments.

*First, experiments assessing ependymal cilia function (Fig. 4D-4G) lack control conditions (e.g. motile cilia paralyzing toxins). These experiments are required to determine whether the measured fluorescent bead speeds reflect the motility of ependymal cilia.*

🡪 We appreciate the comment and agree that this is an essential negative control. In response, we performed the bead motility experiment after 10-min incubation of the brain slices with pneumolysin, a pneumococcal toxin known to inhibit motile cilia in a dose-dependent manner [1, 2]. This treatment significantly reduced the motility of the beads (**Fig 3H**), supporting the validity of the bead motility assay.

*Further, ependymal cilia function at the anterior ventral portion of the lateral ventricle wall, a critical bottleneck of ependymal cilia flow within the lateral ventricles, was not assessed, which would be required for the complete evaluation of ependymal cilia function.*

🡪 In response, we analyzed the bead motility in the anterior ventral portion of the lateral ventricular wall. Similar to the results from the AD/PM areas, the bead motility in the anterior ventral portion was also increased in Katnal2-KO mice (**Fig 3E**).

*Additionally, the authors do not provide sufficient data to support the claim that increased ependymal cilia motility causes an increase in hydraulic pressure. Specifically, the authors report that increased CSF flow rates in the cerebral aqueduct of anesthetized Katnal2 mutants causes an increase in hydraulic pressure (Fig 4J); however, the measurement of intraventricular pressure would be required to provide support for this claim.*

🡪 In response, we measured the intracranial pressure (ICP) in Katnal2-KO mice (3 months), using the pressure sensor previously described [3]. The results indicated comparable ICPs in WT and Katnal2-KO mice (**Fig 3L**). We think that the increased ICP in Katnal-2 KO mice might have been counteracted by the increased ventricular volumes. In line with this, about half of the patients with idiopathic ventriculomegaly show normal ICPs. We commented on this in the revised Results and Discussion.

*The authors show that ventriculomegaly in Katnal2 KO mice is rescued with the delivery of AAV-mediated overexpression of Katnal2 at timepoints before the emergence of hydrocephalus (Fig. 7C); however, analyses of ependymal cilia morphology and function were not performed to validate their hypothesis.*

🡪 In response, we tested if the increased length of ependymal cilia in Katnal2-KO mice could be restored by Katnal2 re-expression and found that the normalization of the ciliary length in Katnal2-KO mice with Katnal2 re-expression to levels comparable to those in wild-type mice (**Fig 6C**).

*Strikingly, the authors show robust endogenous Katnal2 expression within the choroid plexus (Fig. 3C) and remark that this expression may indicate that Katnal2 may regulate the production of CSF; however, no experiments were performed to evaluate a pathological role of the choroid plexus in the etiology of Katnal2-associated ventriculomegaly. This is of fundamental importance because changes in the rate of CSF circulation by ependymal cilia may be secondary to increased production of CSF, not a primary deficit underlying ventriculomegaly.*

🡪 In response, we re-analyzed blood perfusion levels in the choroid plexus. CBV-weighted signals around the choroid plexus were unaffected, (**Fig EV7B,C**), suggesting that CSF production, relying on the blood flow to the choroid plexus [4, 5], was not changed.

*To this point, all of the experiments evaluating ependymal cilia function were performed at timepoints after Katnal2 KO mice have documented ventriculomegaly, and no experiments evaluating ependymal cilia function were done at earlier timepoints when Katnal2 KO mice lack ventriculomegaly (S4A Fig).*

🡪 We appreciate this comment and agree that assessing ciliary function at early stages would improve this manuscript. We have to point out, however, that the full maturation of cilia and their alignments in the ependymal layer has been reported to be finished at a later stage (> postnatal day/P 21) [6]. In addition, Amanda et al. reported that only 26% of ependymal cells are mature (multi-ciliated) at P7, which is increased to ~60% at P30 [7]. In addition, Ohata and Alvarez-Buylla described that the planar cell polarity of multiciliate ependymal cells is established around or after P21, but not at P7 [8]. Moreover, it is known that shearing stress in young brain tissues with low-density cells makes it difficult to prepare healthy brain sections. These aspects discouraged us from performing experiments with slices from younger ages. We pointed out these aspects in the revised Results.

**Reviewer #2,**

*Froylan Calderon de Anda (Note, reviewer 2 has signed this review): This is an interesting and well-executed study that shows that deficiency of Katnal2 is instrumental in progressive ventricular enlargement. Elegantly, authors show that lack of Katnal2 increased the length and beating frequency of motile cilia on ependymal cells lining ventricles; thus, affecting the ventricular hydraulic pressure. Moreover, it is shown that progressive increment of ventricles leads to decrease cerebral blood volumes in various brain regions. Authors suggest that these volumetric changes might affect neuronal functioning and, accordingly, they show progressive synaptic deficits in the hippocampus that are paired with transcriptomic changes. Finally, it is revealed that mice lacking Katnal2 show selective abnormalities in social communication such as ultrasonic vocalizations in the adult and early in the development (pup stage) that are partially overcome upon the ventricular introduction of Katnal2 (postnatal day 8-12).
The paper is well-written, although it could be organized differently with the behavioral analysis at the end together with the rescue approach (my personal taste).  The authors use an interesting approach to test their hypothesis, supplemented with a blend of imaging techniques, and the data is nicely presented.  An interesting new link between cilia functioning and ASD is identified; however, the mechanism by which cilia abnormalities and brain volumetric changes exert their effect on synapse physiology is unclear. Having said this, a number of issues make the interpretation of the results difficult and it would be beneficial if the authors revise their manuscript.*

🡪 We appreciate the encouraging and helpful comments of the reviewer.

1.      I am wondering why the pups show abnormalities in social communication given that the cellular and molecular parameters tested revealed progressive development of anomalies (not present in the pups).

🡪 We agree with the reviewer that the pup USV phenotype may be minimally correlated with the ciliary and ventricular phenotypes. In response and also considering the recommendation of reviewer #3, we decided to remove the pup USV data from the revised manuscript. The remaining ASD-related behavioral data were moved from the main **Fig 1** to a supplementary figure (**Fig EV2**).

*2.      Even though the cellular architecture of the cortex seems to be not affected (cortex layering), suggesting no issues with neurogenesis and neuronal migration, it could be helpful to analyze how neuronal morphology is affected when they lack this katanin-like protein. We know that microtubule dynamics are key during neuronal differentiation. Moreover, neurons have cilia, and little is known about this organelle in neurons. Maybe culturing neurons (or analysis in situ from embryonic tissue) from mice lacking Katnal2 may teach us something that could explain the phenotypes observed in the pups.*

🡪 In response, we performed Sholl analyses to examine the dendritic morphology of Katnal2-KO neurons by crossing Katnal2-KO mice with Thy1-eGFP mice for sparse neuronal labeling. There was no genotype difference in the dendritic morphology of hippocampal CA1 neurons at P21 (**Fig EV1H**). We chose the age of P21 rather than an adult age (i.e. P56) to minimize the confounds induced by the ventriculomegaly evident in adult Katnal2-KO mice.

In addition, we examined the primary cilia in Katnal2-KO cultured hippocampal neurons by immunostaining for the neuronal primary cilia marker Arl13b and the neuronal marker NeuN. We found no genotype difference in the length of the primary cilia in WT and Katnal2-KO mice (**Fig EV1l**). These results suggest that Katnal2 KO has minimal impacts on neuronal dendritic morphology and neuronal primary cilia.

**Reviewer #3:**

*Eunjoon Kim and colleagues present a compelling study that highlights the contributions of the Katnal2 gene in molecular, cellular, and behavioral phenotypes related to Autism Spectrum Disorder (ASD). The authors utilized Katnal2 knockout mice to demonstrate that absence of Katnal2 results in autistic-like behaviors, particularly social communication deficits. These behavioral changes were accompanied by age-dependent ventricular enlargements, elongated ependymal cilia, and increased ciliary beating frequency, which were correlated with the expression of Katnal2 in the ependymal cilia and other brain regions. The study also observed impairments in synaptic functions and transcriptomic signature. Interestingly, re-integration of Katnal2 early postnatally prevented ventricular and behavioral deficits in the adult mice, suggesting a possible link between Katnal2 (dys)function and the observed phenotypes.
The creation of the Katnal2 KO mouse model and the involvement of this gene in the function of the cilia and ventricular dysfunction is a valuable contribution to the scientific community. While the data presented in the study warrant publication in* PLOS Biology*,*

🡪 We appreciate the encouraging and helpful comments of the reviewer.

I suggest removing the behavioural part that does not help to clarify the role of Katnal2 in the functioning of the cilia and brain development. For example at a juvenile age it seems that the social deficits (USV) observed are independent from the ventricules malformation since they are OK.

🡪 In response, we removed the pup USV data from the revised manuscript and moved the remaining ASD-related behavioral data from the main figure (**Fig 1**) to a supplementary figure (**Fig EV2**).

*I am also wondering why the number of mice is quite low for some behaviours and very high for others.*

🡪 As the reviewer correctly pointed out, mouse numbers are smaller in some experiments. This is particularly the case for the bidirectional direct interaction test (B6-B6 pair; n = 6 and 8 for WT and KO mice, respectively), which is because we had to use ‘pairs’ of mice for the experiments. During additional revision experiments, we increased the number of mouse pairs in direct social interaction tests as follows: for B6-B6, 6 and 8 pairs were increased 11 and 13 pairs, respectively, and for B6-C3H, 5 and 7 pairs were increased to 10 and 11 pairs (**Fig EV2B,C**). The results did not change the original conclusions; the lack of genotype differences between WT and Katnal2-KO mice.

**References**

1.	Mohammed BJ, Mitchell TJ, Andrew PW, Hirst RA, O'Callaghan C. The effect of the pneumococcal toxin, pneumolysin on brain ependymal cilia. Microb Pathog. 1999;27(5):303-9. doi: 10.1006/mpat.1999.0306. PubMed PMID: 10547234.

2.	Hirst RA, Rutman A, Sikand K, Andrew PW, Mitchell TJ, O'Callaghan C. Effect of pneumolysin on rat brain ciliary function: comparison of brain slices with cultured ependymal cells. Pediatr Res. 2000;47(3):381-4. doi: 10.1203/00006450-200003000-00016. PubMed PMID: 10709739.

3.	Da Mesquita S, Louveau A, Vaccari A, Smirnov I, Cornelison RC, Kingsmore KM, et al. Functional aspects of meningeal lymphatics in ageing and Alzheimer's disease. Nature. 2018;560(7717):185-91. Epub 20180725. doi: 10.1038/s41586-018-0368-8. PubMed PMID: 30046111; PubMed Central PMCID: PMCPMC6085146.

4.	Lee H, Ozturk B, Stringer MS, Koundal S, MacIntosh BJ, Rothman D, et al. Choroid plexus tissue perfusion and blood to CSF barrier function in rats measured with continuous arterial spin labeling. Neuroimage. 2022;261:119512. Epub 20220723. doi: 10.1016/j.neuroimage.2022.119512. PubMed PMID: 35882269; PubMed Central PMCID: PMCPMC9969358.

5.	Lee D, Le TT, Im GH, Kim SG. Whole-brain perfusion mapping in mice by dynamic BOLD MRI with transient hypoxia. J Cereb Blood Flow Metab. 2022;42(12):2270-86. Epub 20220728. doi: 10.1177/0271678X221117008. PubMed PMID: 35903000; PubMed Central PMCID: PMCPMC9670005.

6.	Guirao B, Meunier A, Mortaud S, Aguilar A, Corsi J-M, Strehl L, et al. Coupling between hydrodynamic forces and planar cell polarity orients mammalian motile cilia. Nature Cell Biology. 2010;12(4):341-50. doi: 10.1038/ncb2040.

7.	Coletti AM, Singh D, Kumar S, Shafin TN, Briody PJ, Babbitt BF, et al. Characterization of the ventricular-subventricular stem cell niche during human brain development. Development. 2018;145(20). Epub 20181026. doi: 10.1242/dev.170100. PubMed PMID: 30237244; PubMed Central PMCID: PMCPMC6215394.

8.	Ohata S, Alvarez-Buylla A. Planar Organization of Multiciliated Ependymal (E1) Cells in the Brain Ventricular Epithelium. Trends Neurosci. 2016;39(8):543-51. Epub 2016/06/18. doi: 10.1016/j.tins.2016.05.004. PubMed PMID: 27311928; PubMed Central PMCID: PMCPMC5312752.
