# Supplementary material for: Loss of Katnal2 leads to ependymal ciliary hyperfunction and autism-related phenotypes in mice
Source: PLoS Biol. 2024 May 8;22(5):e3002596. doi: 10.1371/journal.pbio.3002596 (PMC11104772; doi:10.1371/journal.pbio.3002596)
Supplement: Attachment — Submitted filename: Point by point response Kang et al.docx [file pbio.3002596.s017.docx]

**Point-by-point responses**

**Re:** **PBIOLOGY-D-23-00657R2**

***Reviewer #1****:*

*A recent pre-print has been published by Qiu et al., on BioRxiv (doi:* [*https://doi.org/10.1101/2023.07.03.547302*](https://doi.org/10.1101/2023.07.03.547302)*), delineating the mechanism of ventricular enlargement in a Katnal2 mutant mouse model with conditional deletion of Katnal2 specifically within epithelial cells of the ependyma and choroid plexus of neonatal mice. Qui et al. report that Katnal2 mutant mice have decreased ependymal cilia beating and reduced CSF flow, consistent with previously published findings in Katnal1 mutant mice with ventriculomegaly. These data contradict data presented here by Kang et al. showing increased ependymal cilia beating frequency and increased CSF flow rates despite normal ICP. Major concerns remain about the mechanism of ventriculomegaly, and the recently published pre-print decreases novelty of this finding in Katnal2 mutant mice.
Due to this, my recommended course of action is rejection of this manuscript.*

🡪 We have to point out that the Katnal2-mutant mouse line that lacks exon 8 reported in the preprint publication is ‘qualitatively’ different from our Katnal2-mutant mouse line that lacks exon 3. The exon 8 deletion leads to a small in-frame deletion (aa 220–245; 26 aa residues) in a region immediately preceding the AAA-ATPase domain located in the middle of the Katnal2 protein (539 aa-long; see the figure below), likely leaving the exon 8-lacking Katnal2 protein variant largely similar to the whole protein although it might destabilize the protein, which was not directly tested. On the other hand, our mutation (exon 3 deletion) leads to an out-of-frame protein-truncating mutation in the N-terminal region of the protein at around ~aa 69 (see below), almost completely eliminating the whole Katnal2 protein. The abovementioned preprint publication also reports another Katnal2 mouse line lacking exons 2–12, a larger deletion compared with the exon 8 deletion. However, this mouse line was used to measure ventriculomegaly and choroid plexus-related measures but not other phenotypes that help infer how the ventriculomegaly was induced such as ependymal ciliary functions, making it unclear if the ventriculomegaly observed in these mice involves decreased or increased ependymal ciliary function. These differences and uncertainties should be clarified in future studies. We mentioned these aspects in the revised Discussion.

*The inclusion of a toxin washout experiment to show restoration of ependymal cilia function would have been the proper experiment to validate the bead motility assay.*

🡪 Pneumolysin is a well-known toxin that inhibits ependymal ciliary function by forming pores on the surface membrane, a process that has recently been shown to be irreversible by ATM microscopy (PMID: 27796097, PMID: 28323617). We commented on this with references in the revised results.

*The authors provide data that Katnal2-KO mice have comparable ICPs to WT mice. It is highly unlikely that increased ependymal cilia motility causes increased hydraulic pressure without also raising intraventricular pressure. This mechanism has never been described in mice or humans with ventriculomegaly and is inconsistent with previous findings in Katnal1-KO mice and the recent Katnal2-KO pre-print manuscript.*

🡪 We respectfully disagree that we cannot propose a working hypothesis or an interpretation based on the reviewer’s argument that ‘this mechanism has never been described’. Our hypothesis tries to link ‘increased ciliary length and beating rate’ to ‘increased hydraulic pressure’ to ‘ventriculomegaly with normalized intracranial pressure (ICP) by surrounding tissue shrinkage’. We believe that the ICP could be increased while ventriculomegaly is induced but normalized after the ventriculomegaly reaches a peak perhaps in a steady-state equilibrium. We briefly mentioned this possibility in our revised Results.

*The authors provide data that Katnal2 overexpression restores ciliary length; however, the functional analysis of these cilia with normalized length was not assessed.*

🡪 We have already demonstrated that Katnal2 re-expression rescues the length of ependymal cilia, which, together with the increased ciliary beating rate, has been suggested to cause the ventriculomegaly observed in Katnal2-mutant mice. After a discussion with the editor, we decided not to perform the suggested experiment. We hope that the reviewer understands.

*The authors examine blood perfusion levels in the choroid plexus as a proxy for CSF production; however, this is an inadequate assessment of CSF production. It has previously been shown that the cilia of choroid plexus epithelial cells regulate CSF production. Despite robust Katnal2 expression within the choroid plexus, the authors examined cilia length of ependymal epithelial cells without also examining cilia length of epithelial cells of the choroid plexus. At minimum, the authors should have examined the expression of ion co-transporters (Na+-K+-ATPase, AE2) and water channels (AQP1) in Katnal2-KO mice.*

🡪 We performed the suggested immunoblot analysis and found that there is no genotype difference in the choroid plexus levels of (Na^+^-K^+^-ATPase, AE2, and AQP1) (**Figure EV7D**), further suggesting, together with the lack of changes in blood flow to the mutant choroid plexus (**Figure EV7B,C**), that Katnal2 deletion does not change CSF production in the choroid plexus.

***Reviewer 3****:*

*I appreciate the authors additional experiments added to the manuscript. However, I maintain my viewpoint that keeping the data on social, even to the supplementary section, diminishes the paper's strength by diverting attention from its core focus. I leave it to the discretion of the authors and the editor to decide whether they wish to retain it. Nevertheless, I support the publication of the manuscript, the scientific community will benefit of a new mouse model that could enhance our understanding of one of the various functions of cilia.*

🡪 We appreciate this suggestion. However, we would like to maintain the behavioral data in supplementary figures because we think it is a meaningful data supporting the face validity of Katnal2-mutant mice for ASD, and a related preprint paper is describing behavioral results in the main figure. This would help readers to better compare the two studies.
